# Usability Testing of an Online Self-management Program for Adolescents With Juvenile Idiopathic Arthritis

**DOI:** 10.2196/jmir.1349

**Published:** 2010-07-29

**Authors:** Jennifer Stinson, Patrick McGrath, Ellen Hodnett, Brian Feldman, Ciaran Duffy, Adam Huber, Lori Tucker, Ross Hetherington, Shirley Tse, Lynn Spiegel, Sarah Campillo, Navreet Gill, Meghan White

**Affiliations:** ^10^Department of RheumatologyBC Children’s HospitalVancouverCanada; ^9^Department of RheumatologyMontreal Children’s HospitalMontrealCanada; ^8^Department of RheumatologyIWK Health CentreHalifaxCanada; ^7^Department of ResearchIWK Health CentreHalifaxCanada; ^6^Faculty of MedicineUniversity of TorontoTorontoCanada; ^5^Lawrence S. Bloomberg Faculty of NursingUniversity of TorontoTorontoCanada; ^4^Child Health Evaluative SciencesThe Hospital for Sick ChildrenTorontoCanada; ^3^Department of RheumatologyThe Hospital for Sick ChildrenTorontoCanada; ^2^Centre for NursingThe Hospital for Sick ChildrenTorontoCanada; ^1^Department of Anesthesia and Pain MedicineThe Hospital for Sick ChildrenTorontoCanada

**Keywords:** Juvenile idiopathic arthritis, Internet, usability testing, self-management, adolescent

## Abstract

**Background:**

A new bilingual (English and French) Internet-based self-management program, Teens Taking Charge: Managing Arthritis Online, for adolescents with arthritis and their parents was developed following a needs assessment.

**Objectives:**

This study explored the usability (user performance and satisfaction) of the self-management program for youth with juvenile idiopathic arthritis (JIA) and their parents to refine the health portal prototype.

**Methods:**

A qualitative study design with semi-structured, audio taped interviews and observation by a trained observer was undertaken with two iterative cycles to determine the usability (ease of use, efficiency, errors, and user satisfaction) of the user interface and content areas of the intervention. A purposive sample of English-speaking (n = 11; mean age = 15.4, standard deviation [SD] 1.7) and French-speaking (n = 8; mean age = 16.0, SD 1.2) adolescents with JIA and one of their respective parents/caregivers were recruited from 2 Canadian tertiary care centers. Descriptive statistics and simple content analyses were used to organize data into categories that reflected the emerging usability themes.

**Results:**

All of the participants had access to a computer/Internet at home; however, adolescents were more comfortable using the computer/Internet than their parents. Adolescents and parents provided similar as well as differing suggestions on how the website user interface could be improved in terms of its usability (navigation; presentation and control usage errors; format and layout; as well as areas for further content development). There were no major differences in usability issues between English- and French-speaking participants. Minor changes to the website user interface were made and tested in a second cycle of participants. No further usability problems were identified in the second iterative cycle of testing. Teens and parents responded positively to the appearance and theme of the website (ie, promoting self-management) and felt that it was easy to navigate, use, and understand. Participants felt that the content was appropriate and geared to meet the unique needs of adolescents with JIA and their parents as well as English- and French-speaking families. Many participants responded that the interactive features (discussion board, stories of hope, and video clips of youth with JIA) made them feel supported and “not alone” in their illness.

**Conclusions:**

We describe the usability testing of a self-management health portal designed for English- and French-speaking youth with arthritis and their parents, which uncovered several usability issues. Usability testing is a crucial step in the development of self-management health portals to ensure that the various end users (youth and parents) have the ability to access, understand, and use health-related information and services that are delivered via the Internet and that they are delivered in an efficient, effective, satisfying, and culturally competent manner.

## Introduction

Juvenile idiopathic arthritis (JIA) is the most common rheumatic disease in childhood [1]. JIA can negatively impact all aspects of health-related quality of life (HRQOL) [2]. Disease management is often complex, involving diverse multifaceted therapies over long periods of time that require frequent monitoring. As children mature, they are expected to assume increasing responsibility for disease management concomitant with their growing independence and autonomy [3]. However, adolescents’ acceptance of and ability to implement this increasing responsibility is not always optimal, thereby often reducing the potential benefits of treatment and adversely impacting HRQOL [4]. In the absence of cure, improving HRQOL through better disease self-management becomes critical [3].

Self-management interventions typically encompass educational strategies designed to achieve optimal patient knowledge, understanding, beliefs, and skill acquisition as well as to provide meaningful social support [5]. However, the vast majority of adolescents with JIA do not receive comprehensive disease education linked with self-management strategies due to (1) difficulty accessing these services (eg, no services available in many geographic areas, language barriers, or long-wait times), (2) limited availability of trained professionals (eg, psychologists and nurses) especially in rural and remote areas, and (3) costs (eg, time away from school for students or time away from work for parents) associated with these therapies [6,7]. There is a clear need for developing alternative and acceptable ways to deliver self-management therapies to youth with JIA and their families.

With emerging interactive and communication technologies (eg, eHealth), new media for the delivery of health interventions are now available [8]. The Internet has emerged as one of the top health information resources and mode of social communication for youth and is, therefore, ideally suited to the provision of online health care services to adolescents [9]. Internet interventions are treatments based on effective face-to-face interventions (eg, cognitive-behavioral therapies and psycho-education) that are transformed for delivery via the Internet with the goal of improved health outcomes, such as symptom reduction. Usually, such interventions are highly structured, self-guided or partly self-guided, tailored to the user’s needs, and interactive [8]. While this is a burgeoning field, the Internet remains a relatively new medium for delivery of health care for children [10]. Formal evaluations of the impact of Internet health interventions on health outcomes, level of resource utilization, and user satisfaction have lagged far behind their development [11]. Furthermore, there is a paucity of high quality multilingual Internet health information at an appropriate reading level for youth with JIA and their parents, and no Internet-based self-management program for youth with JIA exists [12].

We developed the Teens Taking Charge: Managing Arthritis Online treatment program. This program is a multi-component, interactive Internet-based program consisting of JIA-specific education, self-management strategies, and social support (in the form of a discussion board and video clips) designed for youth with JIA and their parents that is available in English and French. This program was developed and evaluated using a sequential phased approach consisting of program development, usability testing, and outcome evaluation [13]. In Phase 1, the content of the Internet program was developed based on a needs assessment of adolescents with JIA and their parents [14]. In Phase 2, formative evaluation of the usability of the Internet program was tested and is the focus of this paper. In Phase 3, the feasibility of the intervention was recently evaluated in a pilot randomized controlled trial prior to conducting a large-scale multicentered trial to determine the program’s effectiveness [15].

Usability testing is a critical step in the development of Internet interventions and solicits end-user feedback to learn what works, what doesn't, and where gaps in information or functionality exist using iterative cycles to refine the prototype [16,17]. These factors may impact the frequency of use, understanding, and the likelihood that a user will implement the recommendations [8,16,17]. Usability testing can also help determine the appropriateness of the website interface and content [18], especially when it is designed for different audiences (eg, youth and parents) and delivered in different languages. The usability of the Teens Taking Charge: Managing Arthritis Online treatment program was assessed in terms of user performance (ease of use, ease of learning, errors, and efficiency) and satisfaction with content, user interface, and functionality of the program.

## Methods

### Patient Selection

Adolescent/parent dyads were recruited from 2 large rheumatology clinics in university-affiliated pediatric tertiary care centers. Dyads were eligible to participate if (1) the adolescents were 12 to 18 years of age, (2) the adolescent had been diagnosed with JIA by a rheumatologist, and (3) both adolescent and parent were able to speak and read English or French as their primary language. Adolescents were excluded if they had major cognitive impairments or comorbid medical or psychiatric illnesses that may have impacted their ability to understand and use the Internet program. Eligible patients listed on the participating hospitals’ rheumatology database registries were sent a study information letter inviting them to participate. This method was supplemented by convenience sampling of patients attending a regularly scheduled clinic visit to achieve a heterogeneous sample in terms of age and disease severity. The study was approved by research ethics boards at both sites.

### Study Design and Procedures

A qualitative usability testing approach with semistructured, audio taped interviews and observation by a trained observer was undertaken, with iterative cycles to determine the usability of the Internet intervention and to further refine the prototype [16,17]. This design approach was based on the concept of a ‘‘hermeneutical circle’’ as described by Snodgrass and Coyne [19], which is an iterative process of implementing a design, learning and understanding from discussion and feedback, and making subsequent design refinements. This iterative rapid design development approach concentrated on testing the user-interface with a focus on user performance and satisfaction.

After giving informed consent, participants were asked to complete a general information questionnaire that included questions about demographic characteristics and level of comfort with computers and the Internet. A health information questionnaire was completed by a research assistant to gather additional demographic and disease-related data from the patient’s chart (duration of illness, disease subtype and the rheumatologist’s global assessment of the patient’s disease severity rated on 10-cm visual analogue scale) [20]. Adolescents and their parents then participated in separate 30- to 45-minute semistructured usability testing interviews in a quiet room with computer and Internet access. Participants were given a brief explanation of the online program by the research assistant. The sessions were audio taped and participants were asked to “think aloud” as they worked through a “live” version of the program. Participants were guided to move through a standardized list of features in each of the 12 modules, which included content, graphics, video clips, interactive components, animations, and the discussion forum, while commenting on any difficulties they had. It was anticipated that participants would be able to move through all of these tasks during the usability interview (ie, within 40 minutes). The research assistant also recorded the length of the sessions and made field notes about any problems encountered during the sessions (eg, tracking participant navigation errors and program errors) as well as ease of use and learning. At the end of the session, participants were asked a series of questions using a semistructured interview guide to ascertain user satisfaction and suggestions for improving the program (See Multimedia Appendix 1). Interviews were conducted in two iterative cycles, one cycle per site. Following the first iterative cycle with English-speaking participants, changes were made to the user interface screens based on problems identified from the content analysis of the taped interviews and field notes. The revised user interface was then evaluated in a second iterative cycle with French-speaking participants.

### Internet Self-management Program

The Internet program is a 12-module interactive multi-component treatment protocol that consists of JIA-specific education (eg, common problems associated with treatment and disease), self-management strategies (eg, how to deal with pain and stress), and social support (eg, monitored discussion boards and narratives in the form of written stories and video clips of adolescents with JIA). There are two modules specifically for parents to help them promote healthy teens’ behaviors. The program content was developed by a team of experts from across Canada, written at a grade 6 to7 reading level, and geared to the needs identified by adolescents and their parents [14]. The Teens Taking Charge: Managing Arthritis Online treatment program is composed of 310 content pages (approximately 156459 words), flash files (animations), images, videos, forums (separate adolescent and parent discussion boards, surveys, and interactive forms (eg, weekly knowledge quizzes). The entire program was translated into French using forward and backward translation as well as being reviewed by Francophone medical experts. The video clips of teens for the French site were recorded with Francophone adolescents and their parents in Montreal to ensure they were culturally competent.

In the website architecture, four applications are integrated together to provide the experience of a single website to the users. These applications include (1) the content application for both English and French, which is delivered by the AboutKidsHealth.ca content management system; (2) the forums application, which provides a framework for creating and managing discussion forums for the users; (3) the survey application, which provides support for creating, managing, and gathering survey questions and responses; and (4) the custom application, which provides management of interactive questions. Each of the applications has its own database model where data are managed and stored. Security and restriction of the program are based on the ASP.NET membership model and ensure that only authenticated users with the allowed permission (ie, role) are able to view the allowed content and to participate in the private forums. The membership model works across several applications and accommodates a single sign-on process so users do not have to sign in to every application and thus can move across the applications seamlessly. Users get the best experience of the Teens Taking Charge: Managing Arthritis Online treatment program with Internet Explorer 6/7 and Flash player 9+ on a Windows XP/Vista platform. The given computer requirements ensure that users are able to view the animation and video streams within the site and as well view the embedded interactive forms, which are delivered via an AJAX interface that captures the responses to a database. The AJAX interface reduces the number of clicks and pages to keep the user experience intuitive. Please see Multimedia Appendix 2 for an overview of the Teens Taking Charge: Managing Arthritis Online progam.

### Data Analysis

The quantitative data from the questionnaires was analyzed using SAS version 9.1.3 [21] to determine measures of central tendency and the distribution of values. Interviews were audio taped and transcribed verbatim. The French interviews were transcribed directly into English by two bilingual transcriptionists. Field notes taken during the interviews were also transcribed and included in the analytic process. Simple content analysis was performed after each iterative cycle, with categories emerging form the usability research questions (ie, learnability, efficiency, errors, and satisfaction) and frequencies calculated [22]. Initially, the English (iterative cycle 1) and French (iterative cycle 2) interviews were analyzed separately. Minor modifications to the website were made based on the first iterative cycle of testing and were subsequently evaluated in the second cycle of testing. No further modifications to the prototype were made based on the second iterative cycle of testing. The data from these two cycles were similar and provided a strong source of triangulation for the developing themes. To ensure anonymity, all participants were identified by pseudonyms, and quotes were identified by participant type (adolescent = A; parent = P) [23].

## Results

### Sample Selection and Participant Characteristics

Patients meeting the study criteria who attended clinics from August 22 through October 2, 2008, were invited to participate in this study at the two sites. Of the 20 eligible English-speaking dyads, 11 (55%) agreed to participate, and of the 23 French-speaking dyads, 8 (39%) agreed to participate. The mean age of the sample of adolescents was 15.7 years (SD 1.5), and 14 (74%) were female. Of the 19 adolescents, 15 (79 %) were in high school. The mean duration of illness was 6.99 years (SD 5.03, range 0.3 - 15.2 years). The most common disease onset subtype was polyarticular arthritis with 7 of the 19 adolescents (37%) having this diagnosis; the average disease severity rating was 0.92 out of 10 (SD 1.5, range 0 - 4.9). Of the participating adolescents, 100% had a computer at home, and 100% were comfortable using a computer. These findings are in accordance with recent data that 88% of 15-year-old Canadian students have at least one computer at home and are comfortable using the computer/Internet [24]. Thirteen of the 19 parents/caregivers were female (63%), 10 of the 19 parents/caregivers (53%) were aged 40 to 49 years, and 9 (47%) responded that their highest level of education was high school. The numbers, means, and standard deviations for demographic and disease-related characteristics of the adolescent sample by usability testing phase are shown in Table 1. Participants’ comfort level with and use of computers and the Internet are described in Table 2.

**Table 1:** Characteristics of the adolescents from interative cycles 1 and 2 (n = 19)

Characteristic		Iterative Cycle 1 English (n = 11)	Iterative Cycle 2 French (n = 8)
Mean age (SD) in years		13.6 (2.03)	16 (1.23)	
**Gender**				
	Male, n (%)	3 (27%)	2 (25%)	
	Female, n (%)	8 (73%)	6 (75%)	
**JIA onset subtype**				
	Oligoarthritis, n (%)	1(9%)	2 (25%)	
	Polyarthritis (RF -), n (%)	4 (36%)	3 (38%)	
	Polyarthritis (RF +), n (%)	2 (18%)	0	
	Psoriatic arthritis, n (%)	1 (9%)	0	
	Enthesitis related, n (%)	1 (9%)	1(13%)	
	Systemic, n (%)	0	2(25%)	
	Unknown, n (%)	2(18%)	0	
Mean (SD) duration of illness in years		7.23 (4.45)	6.65(6.05)	
**Current grade**				
	Public school, n (%)	2 (18%)	0	
	High school, n (%)	8 (73%)	7 (88%)	
	University, n (%)	1 (9%)	1 (12%)	

**Table 2:** Information about use of the computer by adolescents and their parents

		Adolescents		Parents	
		English (n = 11)	French (n = 8)	English (n = 11)	French (n = 8)
Characteristic		n (%)	n (%)	n (%)	n (%)
**Computer at home**					
	Yes	11 (100)	8 (100)	11 (100)	8 (100)
	No	0	0	0	0
**Internet at home**					
	Yes	11 (100)	8 (100)	11 (100)	8 (100)
	No	0	0	0	0
**Computer at school/work**					
	Yes	9 (82)	5 (63)	9 (82)	6 (75)
	No	1 (18)	3 (37)	1 (18)	2 (25)
**Hours spent on computer each week**					
	≤ 5 hours	1 (9)	3 (37)	3 (27)	3 (37)
	> 5 hours	10 (91)	5 (63)	8 (73)	5 (63)
**Hours spent on Internet each week**					
	≤ 5 hours	4 (36)	3 (37)	7 (64)	5 (63)
	> 5 hours	7 (64)	5 (63)	4 (36)	3 (37)
**Comfort level on computer**					
	Not at all comfortable	0	0	0	1 (12)
	A little comfortable	0	0	2 (18)	0
	Comfortable	2 (18)	0	4 (36)	2 (25)
	Very comfortable	9 (82)	8 (100)	5 (45)	5 (63)
**Comfort level on Internet**					
	Not at all comfortable	0	0	0	1 (12)
	A little comfortable	0	0	2 (18)	0
	Comfortable	2 (18)	0	4 (36)	2 (25)
	Very comfortable	9 (82)	8 (100)	5 (45)	5 (63)

### User Performance

User performance was measured in terms of (1) ease at which the user could navigate through the site, (2) ease of learning in terms of how fast a user who has never seen the user interface before could learn it sufficiently well to accomplish basic tasks, and (3) the frequency and severity of errors. Overall the participants rated the Teens Taking Charge: Managing Arthritis Online health portal as easy to use. All of the adolescents were able to navigate through the site with little to no guidance, whereas 5 of the 19 parents (27%) required several minutes to orientate themselves to the site before confidently navigating the interface. In terms of ease of learning, all participants were able to accomplish the standardized set of basic tasks in 35 minutes or less, as was predicted.

**Figure 1:**
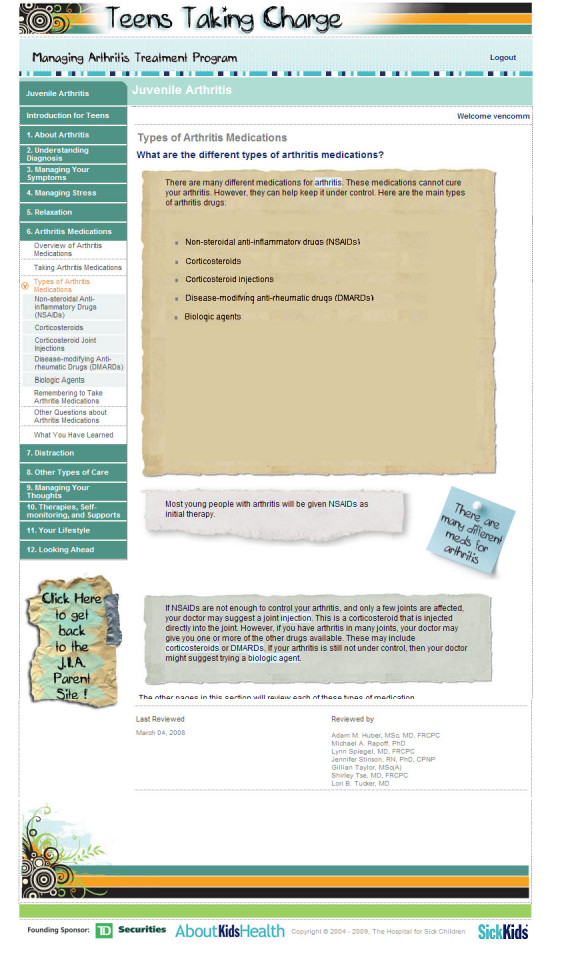
Example of medication homepage before usability testing

**Figure 2:**
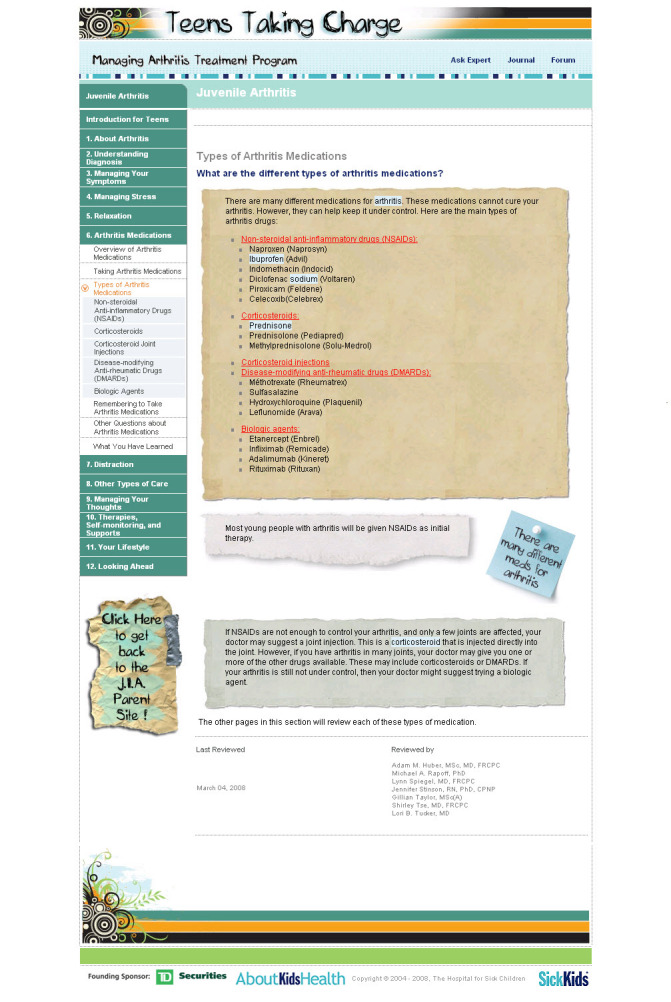
Example of changes to medication homepage following iterative cycle1 testing

**Figure 3:**
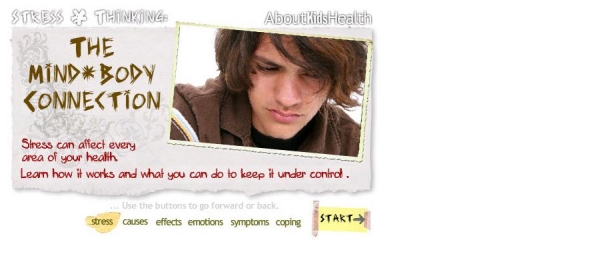
Example of a “stress and thinking” click through animation

During the usability testing, three types of errors were observed: navigation errors, presentation errors, and control usage problems. Navigation errors were defined as failures to locate functions, excessive keystrokes, or failures to follow recommended screen flow. There were minimal navigation errors with 10% of adolescents and 21% of parents experiencing one. All navigation errors encountered were easily recovered from and were considered to be minor. No changes were made based on these findings. Presentation errors were defined as failures to locate and properly act upon desired information or selection errors due to labeling ambiguities. These were found to be concentrated in the medication module. Presentation errors were encountered by 58% of parents and 26% of adolescents on the homepage of the medication module, which were coded as fatal errors. Users found the labeling on the medication homepage by classification of medication type to be unclear and were unable to navigate to the information they wanted about their/their child’s particular arthritis medication(s). This page was changed by adding a list of generic medications grouped by their drug classification with direct links to each medication discussed in that section to enhance user performance (See Figures 1 and 2 for before and after screen shots of medication homepage). Following this change, this presentation error did not occur in iterative cycle 2 testing with French-speaking participants. Control usage errors were defined as improper tool bar or entry field usage. In iterative cycle 1, 42% of adolescents and 26% of parents were unable to identify controls to stop, start, or advance the functional features on the “stress and thinking animation” and video clips at the start of each module, which were coded as fatal errors. These were changed and retested in iterative cycle 2 with no further control usage errors arising. See Figure 3 for an example of the “stress and thinking” animation controls following usability testing. There were no differences between French and English participants in the types of errors found.

### User Satisfaction

In terms of satisfaction with the Teens Taking Charge: Managing Arthritis Online health portal, adolescents and their parents perceived that their satisfaction with the site was based on the overall design aesthetics of the user interface, content, functionality and features, and opportunities for social support. There were minor differences within these categories by participant (adolescent versus parent/caregiver) and language.

### Design Aesthetics

The overall design aesthetics of the website were felt to be a critical factor from the perspective of the end users that helped to enhance engagement and motivation to use the site. Four subthemes were identified under the broad theme of design aesthetics including layout, navigation, visual assets, and visual appeal. Differences were found between both adolescents and parents and French and English participants. Adolescents wanted the large amounts of text “chunked up” and broken up with visual assets (graphics, animations, illustrations), while parents did not mind having large amounts of text to scroll through. Similarly, 42% of parents indicated that they would like a larger font; however, only 15% of adolescents cited this as a problem. Furthermore, 16% of both French adolescents and parents suggested adding labels to the medical diagrams, where this was not an issue with English participants. Finally, all the participants felt that the most important information on each page should be at the top of the page, otherwise teens will not scroll down to find it. See Table 3 for a summary of issues and changes made to the design aesthetics.

### Content

All of the participants commented positively on the content of the program including the text, images, and multimedia components. Four subthemes were identified under the broad theme of content including completeness, understandability, quality and credibility, and relevance. For the most part, participants were satisfied with the completeness of content; however, 21% of adolescents and 32% of parents suggested additional content. The most common suggestion for additional content was for videos or photos to show teens how to do the various exercises. However, due to budgetary and time constraints we were not able to add these. Additionally, 89% of adolescents and 95% of parents felt that the content was relevant, and 74% of adolescents and 79% of parents rated the content as trustworthy. The site was created with a focus on understandability and all text was developed at a grade 6 to7 reading level. Participants expressed appreciation for this consideration and generally found the information, language level, and explanation of medical terminology (in a glossary of words) to be helpful in furthering their understanding of topics that were either unclear, new to them, or both. French grammar errors were found by 20% of French adolescents and 5% of French parents. These errors were corrected following usability testing. See Table 4 for a summary of comments and changes made with regard to website content.

### Functionality and Features

Functionality and features refers to the adaptive and interactive features of the website. These included a glossary of terms, relaxation audio clips, printable PDF information forms for teachers, click-through animations (ie, how we feel pain, how antiinflammatory medications work, stress, and thinking), videos of teens with arthritis, interactive questions (weekly knowledge quizzes), a journal for keeping track of symptoms and weekly goals, and a discussion board with an “Ask an Expert” feature where participants could send an email question to a designated health care professional. Participants unanimously felt that these adaptive and interactive features allowed for an increased level of personalization of the website content to meet the individual needs of the users and were all good features of the website. They also commented that these features would help to enhance motivation and engagement with the program.

**Table 3:** Summary of user satisfaction with design aesthetics and changes made to the site

Design Aesthetics	Examples of Comments by Adolescents	Examples of Comments by Parents	Subsequent Changes Made to the Site Based on Comments
Layout: the compilation and placement of text and graphics on each page	“I really like the layout. I really like how it looks; I find it easy to go through everything.” (A05) “It’s good because it is…visually broken up so it doesn’t seem like a big block of text, but it’s still giving you everything you need to know.” (A04) “Yeah, if they just moved this part up [session goals] and they could do like half above the video and then they would know to like keep going after the video.” (A10)	“I don’t think it is too much. I am always of the opinion....the more information for me the better.” (P08) “Like this, it looks like a lot of stuff to read, and I probably, as a teenager…wouldn’t take the time to read it.” (P11) “I’m not sure how important these bottom links are…but if they are, I would bring them up…that is the only thing because you don’t really see them.” (P01)	- Moved session outlines to appear before the video clip at the start of each module - Moved the discussion forum, “Ask an Expert”, and “My Journal” tool bar from the bottom to top of the page - chunked up the text more in the teen introduction module
Visual assets: the illustrations, graphics, and animations in the website	“Oh, I liked the animation [about] taking drugs, and what arthritis is because it showed me what happens and I didn’t know that”(A11) “Alright, the only thing is that if you hadn’t told me I wouldn’t have seen the next and back buttons [stress animation].” (A01)	“They don’t know what’s happening because they never see their x-rays, that’s a great idea.*”*(P04) “I don’t think it [x-ray image] is necessarily clear, for someone who has medical knowledge, yes it works. But otherwise it is difficult.” (P03F)	-Labeled the images of the normal x-rays and MRIs more clearly so users could better understand the differences between images of diseased and normal joints. -Highlighted the control buttons more clearly on the stress and thinking animation so users could stop it if they wanted to.
Visual appeal: the overall look and feel of the website	“User friendly and inviting. I liked everything. It’s very jolly; it doesn’t look too scientifical.”(A04) I’d have to say it would be these pictures…as soon as–like when I walked in, right, I saw that, and I’m like, ‘wow, that’s fairly depressing’…like I look at her and she doesn’t look happy…she doesn’t look like she’s gotten over this.” (A11)	“Oh. I think its dynamite. I really can’t say enough positive things about it. It’s very impressive.” (P08) “Here, for example, I find that [font] hard to read. Maybe make it a bit bigger.” (P06)	-Photo of teen was replaced on the homepage with a more upbeat looking teen. -Not able to increase font as the health portal needed to conform to the hosting site’s (AboutKidsHealth.ca) design suite which features a 12pt Trebuchet, Arial, sans-serif font.
Navigation: the ability of the end user to easily move around the site to find the information they were interested in	“Everything that came up was really well labeled, and you know what you were going to be reading, and you could choose because everything was broken down.” (A04) “It’s pretty simple…it’s easy to find what you want to look at.” (A05)	“It—it works like every kind of website where you’re looking stuff up. You know it’s really simple.” (P03) “I know, um, the napr—what the naproxen is that she takes. But I wouldn’t have a clue where to look under Avara, or methotrexate... like I wouldn’t even know which one of those it is. So, I think…if you could list some of them like examples, or something.” (P05)	- The medication section was changed as per the user performance section.

**Table 4:** Summary of user satisfaction with website content and changes made to the site

Content	Examples of Comments by Adolescents	Examples of Comments by Parents	Subsequent Changes Made to the Site Based on Comments
Completeness: the extent to which the website content contained all desired information	“It’s all here. It’s all on one site. You don’t have to travel for one thing, and then travel for the next thing…It has all the information that you need basically.” (A02F)	“So I think it could possibly be a good one-stop kind of place to go, as opposed to, you know, the times I’ve had to sit down for hours and go to a million different sites looking for different little bits of things.” (P08)	-Participants recommended adding videos of how to do the suggested exercises; however, due to budgetary and time constraints, we were not able to add the exercise videos.
Understandability: the aspects of the content of the website such as readability (reading level), use of plain language, translation of material into French, and explanation of medical terminology	“No, that’s another good thing. They are not huge words that are going to take me forever to look up and then go back on it.” (A09)	“I liked the way things were explained. It was simple and easy to understand.” (P10)	-During the 2^nd^ iterative cycle, French-speaking participants identified some minor grammatical errors in the translation of the English content that were corrected following the second cycle of testing. These minor changes were checked with the bilingual medical expert.
Quality and credibility: the extent to which the participants perceived the content to be accurate and trustworthy	“I find it reassuring that it’s not coming from 1984, and I know that it is current and up to date.” (A06F)	“The volume of credible information in an easy to access package is really impressive. I love that.” (P03)	-No changes were suggested.
Relevance: applicability of the content of the site to the needs of teens with JIA and their caregivers	“Yeah, I think that the stress and the relaxation, I think I would use that a lot because half the time I don’t even know what to do when I am in pain or anything, right?...Right, and I know that when I am stressed out my arthritis does get worse.” (A05F)	“This is bang on, with what we deal with constantly.” (P10)	-No changes were suggested.

### Sociability

Sociability refers to the ability of the Internet program to support social interactions among participants. The site featured 2 discussion boards, one for teens and one for parents, video clips of teens talking about ways they have learned to manage their arthritis, in addition to written stories of hope submitted by teens with JIA. All of the participants commented that these features of the site would help to ease feelings of hopelessness and particularly isolation. However, while almost all teens (95%) indicated that they would like to use the discussion forum to chat with other teens; less than one-half (42%) of parents felt they would use a parent discussion forum. Many teens commented that they did not know another teen with arthritis and that the interactive features (discussion board, stories of hope, video clips of teens with JIA) made them feel supported and “not alone” in their illness.

### Desire to Use Program in the Future

Overall, the program was well received, and 84% of all participants expressed a desire to use the program in the future. Most participants felt the program would have been especially helpful when they were first diagnosed with JIA. Adolescents and caregivers also liked that the site was geared toward the promotion of self-management skills to reduce symptoms, improve quality of life, and promote transition to adult health care services.

## Discussion

This study assessed the user performance and satisfaction with a new health portal designed to help youth with arthritis learn how to better manage their chronic health condition. This health portal is unique in that it was developed for two different end-user groups (youth and their parents) and in two languages (English and French). Testing uncovered user performance errors (minor navigator and major presentation and control usage errors) as well as areas to enhance user satisfaction. Adolescents and parents provided ideas on how the website user-interface could be improved in terms of its usability (navigation, design format and layout, and content). Changes were made to the health portal that corrected the errors uncovered and improved user satisfaction. Formative usability testing was a crucial step to help ensure the relevance of the content and that the website was easy to use and learn, efficient to complete, and acceptable to youth and their parents. The results of this study also provide a model for formative evaluation of other online interventions for delivery of chronic disease self-management, especially those that target different end users and are provided in multiple languages.

This study also highlights the value of conducting usability testing prior to wide scale outcome assessment (ie, a randomized controlled trial to test effectiveness of the program) and/or implementation to ensure that the end product is usable and acceptable to the end users (youth and parents), and is culturally competent when targeting different languages. While there is increased attention regarding the need for health care professionals to provide face-to-face culturally competent care, there is little research examining how to provide culturally competent health interventions delivered over the Internet. Culturally competent care is defined as the ability to effectively work within the cultural context of the client [25]. While only a few differences were found in this study between French- and English-speaking users, it remains imperative that we evaluate the usability of health portals to ensure they include linguistically and culturally appropriate advice, written information, graphics, and other interactive features [26]. Further research is needed to define what is meant by culturally competent Internet-based interventions and how to evaluate these aspects of usability in formative evaluations given the ethnically diverse populations that are affected by this chronic illness.

Furthermore, these types of interventions have typically been developed by health care professionals (ie, a top down approach) with little input from the end users during intervention development. This is particularly important given the proliferation of Internet-based interventions and the resources currently being invested in health-related websites by governments worldwide [27]. Many of these interventions are often of poor quality and of unknown effectiveness [28]. For example, during the development of our website we undertook a descriptive study to determine the quality and content of English language Internet information about JIA from the perspectives of consumers and health care professionals [12]. Most sites targeted parents, and none were specifically developed for youth with JIA. The overall quality of website information was fair, with a moderate level of accuracy; however, the material was written well above the recommended grade level (6 to 8) indicating that the material was difficult to read. Participants in this study commented on the quality and comprehensiveness of the content as well as on the ease of use and the ease of learning the program. Therefore, it is essential that the end users be actively involved in the development and testing of these health portals.

Usability testing also uncovered some differences by end user. However, little is known about how user traits, which refer to user characteristics such as computer proficiency and demographic and disease characteristics, may affect the use of these health portals [29]. As an example, a study by Jesdanun suggested that among people with chronic diseases, being older and less educated were two traits linked to lower Internet usage [30]. Future studies might investigate user traits that predict usability and treatment outcomes with Internet-based health interventions.

A few potential limitations of our study need to be addressed. First, the study was conducted in two tertiary pediatric care centers and the sample was relatively small, which could be considered a threat to the generalizability of the results. However, with a representative sample of end users, typically involving as few as five participants per cycle, the majority of usability problems and issues can be identified. It has been estimated that a single initial cycle of design/evaluation/redesign can lead to as much as a 10-fold reduction in usability problems [18,31]. Second, while several frameworks have been proposed for evaluating information and communication technologies in health care, there is no consensus on the best approach to use [15,29]. However, the rigor of this study was enhanced by using analyst triangulation (ie, using several researchers to analyze the data). Third, the sample was too small to allow us to examine whether factors related to participants (eg, age, gender, and grade level), disease (severity and duration of disease), or experience (access to and comfort with computers and the Internet) were related to the usability of the Internet program. Future work in this area is needed to determine how these user trait characteristics impact usability and outcome assessment. Finally, due to financial and time constraints we were unable to make all of the suggested changes to the health portal prototype.

This study provides strong initial support for the usability of the Teens Taking Charge: Managing Arthritis Online treatment program for youth with JIA. Findings from this study were used to refine the website prototype prior to conducting a pilot randomized controlled trial to determine the feasibility of the Internet-based self-management program for teens with JIA. If effective in improving health outcomes, this program could be used as a template for other pediatric chronic illness interventions as the psychosocial challenges facing adolescents with JIA are generalizable to all youth with chronic illnesses. Furthermore, the Internet may be critical to improving the accessibility and acceptability of self-management programs for the large population of youth with chronic health conditions that are not able to receive these treatments in their local communities. Usability testing during the formative phase of developing Internet-based treatments is a crucial step in ensuring that these interventions are effective and acceptable to youth with chronic health conditions and their parents.

## Multimedia Appendix 1

Teens Taking Charge: Managing Arthritis Online semi-structured interview guide

## Multimedia Appendix 2

Overview of the Teens Taking Charge: Managing Arthritis Online program

## Abbreviations

HRQOL: health-related quality of life
JIA: juvenile idiopathic arthritis
