# Supplementary material for: Usability Testing of an Online Self-management Program for Adolescents With Juvenile Idiopathic Arthritis
Source: J Med Internet Res. 2010 Jul 29;12(3):e30. doi: 10.2196/jmir.1349 (PMC2956330; doi:10.2196/jmir.1349)
Supplement: Supplementary file 1 [file jmir_v12i3e30_app1.doc]

Multimedia Appendix 1. Teens Taking Charge: Managing Arthritis Online semi-structured interview guide

Now that you have used the Teens Taking Charge: Managing Arthritis On-line website, we are interested in learning about what you liked and disliked about the website.

Question:

1. Can you tell me what you liked best about the website?

Probes: *information,* *layout, animations, graphics, video clips, discussion board etc. Can you tell more about that?*

2. Can you tell me what you liked least about the website?

Probes: *information,* *layout, animations, graphics, video clips, discussion board etc. Can you tell more about that?*

3. Can you tell me about how easy it was to navigate or our find your way around the website?

Probes: *What were the challenges of navigating through the site? What would make it easier to navigate through the site?*

4. Can you tell me about what you thought about the overall look of the website?

Probes: *For example, the design, colours, and images on the web-site. Does the website feed warm and friendly or cold and technical? Can you tell me more about that? Do you think it is visually appealing? What would make the web-site more appealing?*

5. Can you tell me what you thought about the information provided on the website?

Probes: *How do you feel about the accuracy of the information? How do you feel about the trustworthiness of the information provided? What do you think about the amount of information that was provided? Was there any information that you thought should be on the site but was not there? Was the information provided helpful? Was the information on the site clearly presented? Was it easy to read and understand? Do you think this information will help you better manage your arthritis (your child’s arthritis)?*

6. Can you tell me about whether or not you would use such a site to learn how to better manage your arthritis? (or to help your child better manage their arthritis)

Probes: *What would motivate you to use the site? What would make it easier to motivate other teens (parents) like you to use the site?*

7. Can you tell me about whether or not you think other teens (parents) would be interested in using this site to learn how to better manage their arthritis (their child’s arthritis)?

Probes: *What would interest them in using this site? Would you recommend this site to a friend with arthritis? Can you tell me more about that?*

8. If you could make changes to the website, what changes would you make?

Probes: *Can you tell me more about that?*

9. Is there anything else you would like to tell us the website?

Probes: *Can you tell me more about that?*
