# Supplementary material for: Usability Testing of an Online Self-management Program for Adolescents With Juvenile Idiopathic Arthritis
Source: J Med Internet Res. 2010 Jul 29;12(3):e30. doi: 10.2196/jmir.1349 (PMC2956330; doi:10.2196/jmir.1349)
Supplement: Supplementary file 2 [file jmir_v12i3e30_app2.pptx]

## Slide 1
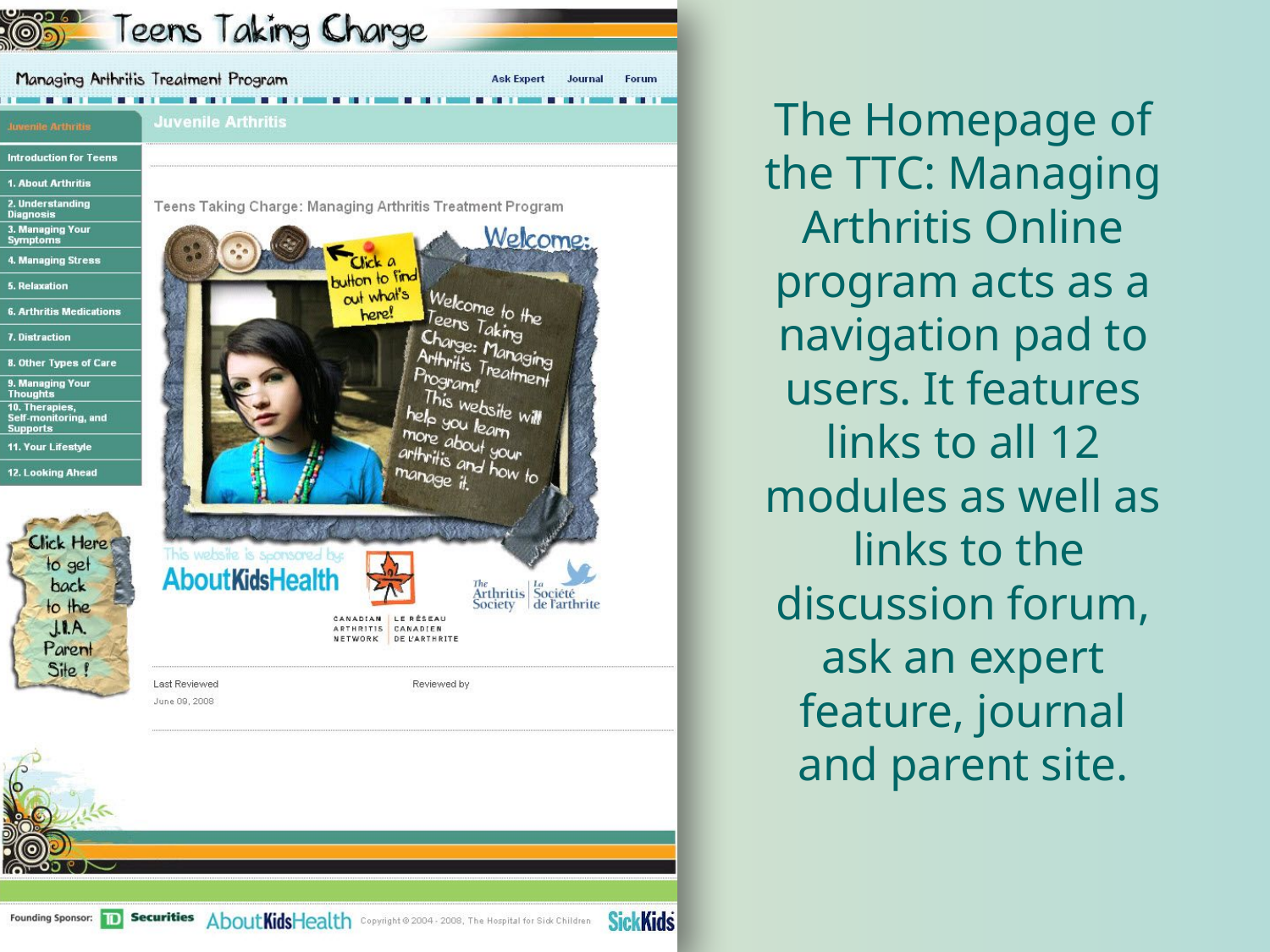

The Homepage of the TTC: Managing Arthritis Online program acts as a navigation pad to users. It features links to all 12 modules as well as links to the discussion forum, ask an expert feature, journal and parent site.

## Slide 2
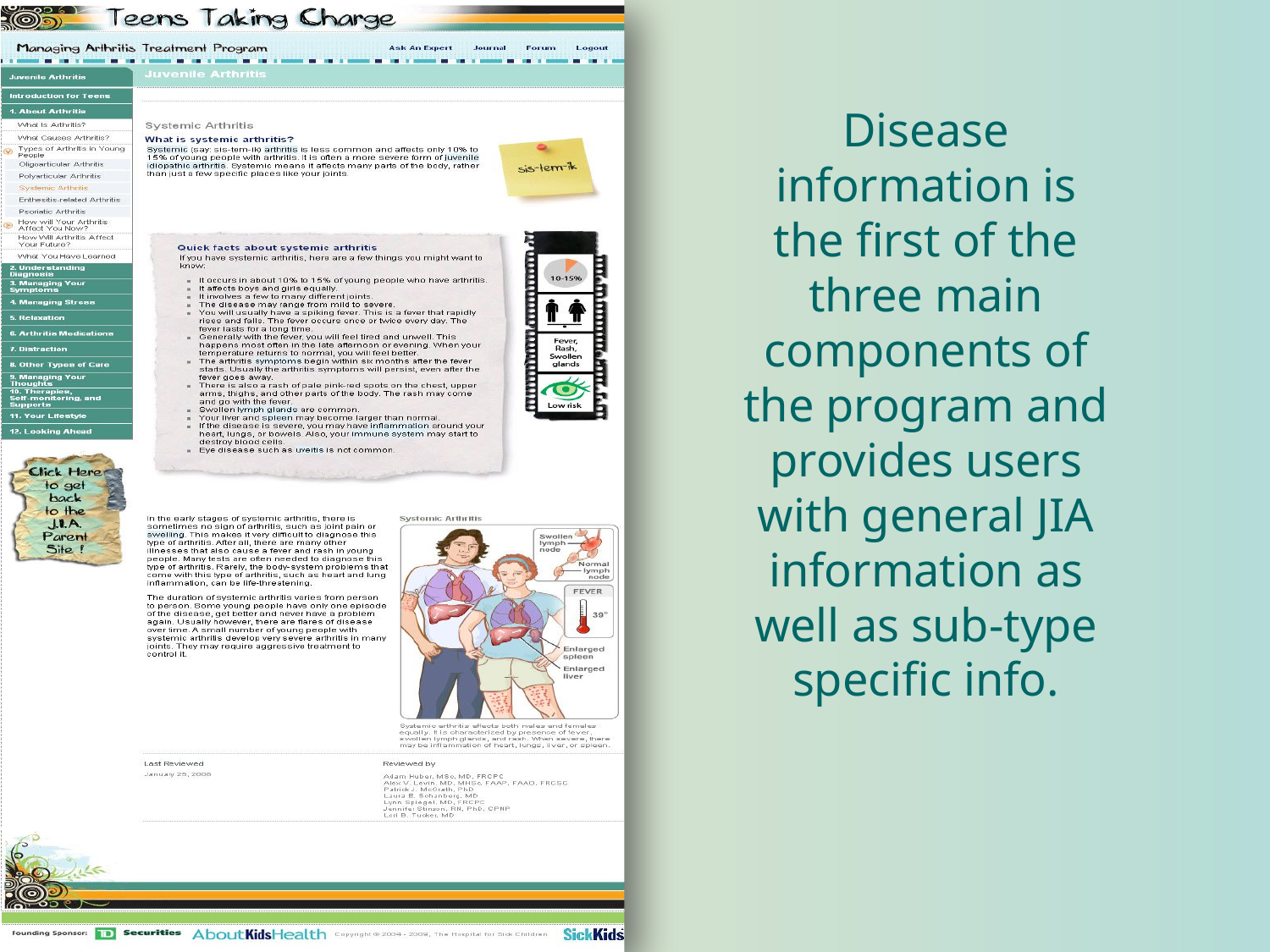

Disease information is the first of the three main components of the program and provides users with general JIA information as well as sub-type specific info.

## Slide 3
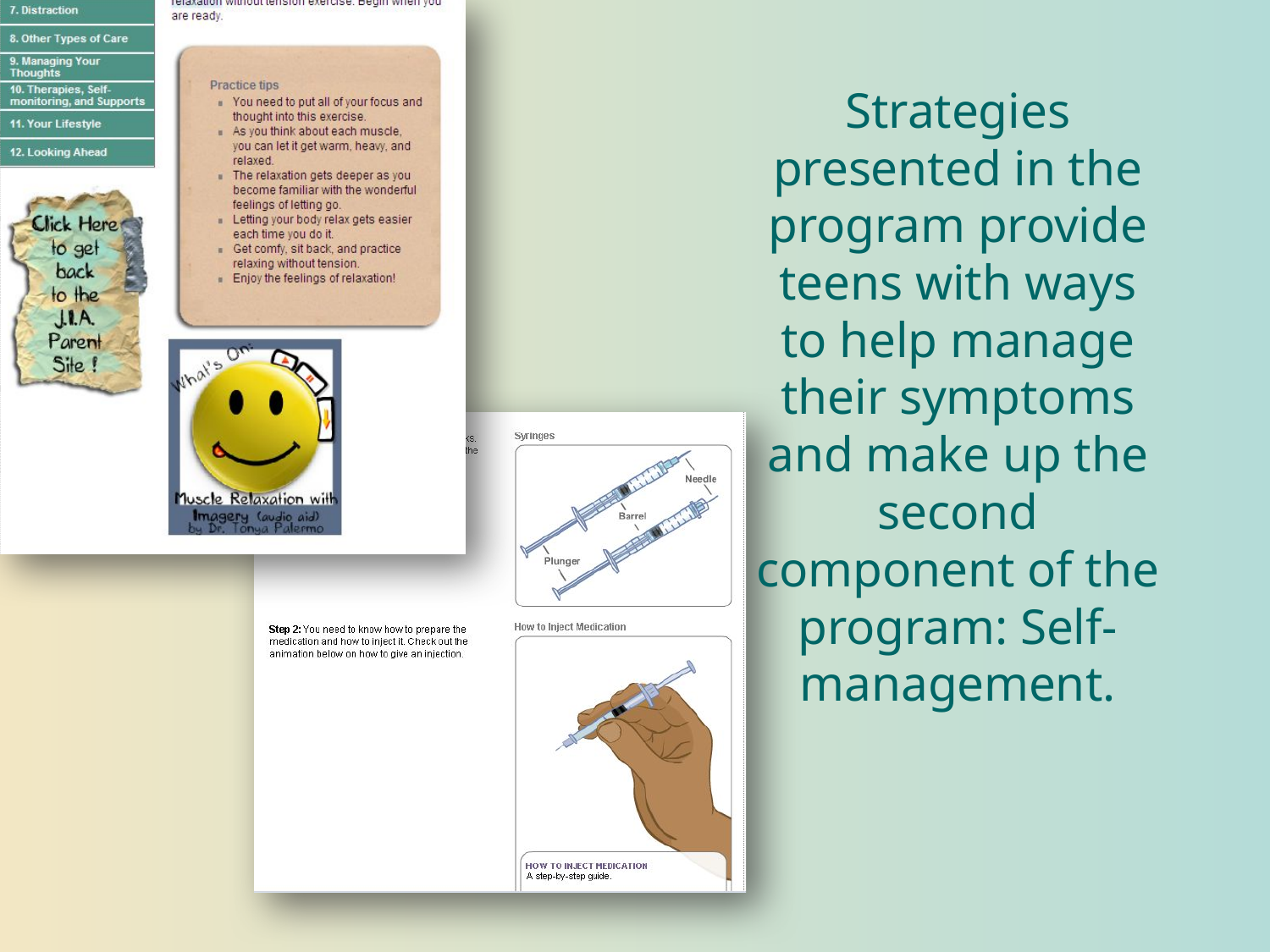

Strategies presented in the program provide teens with ways to help manage their symptoms and make up the second component of the program: Self-management.

## Slide 4
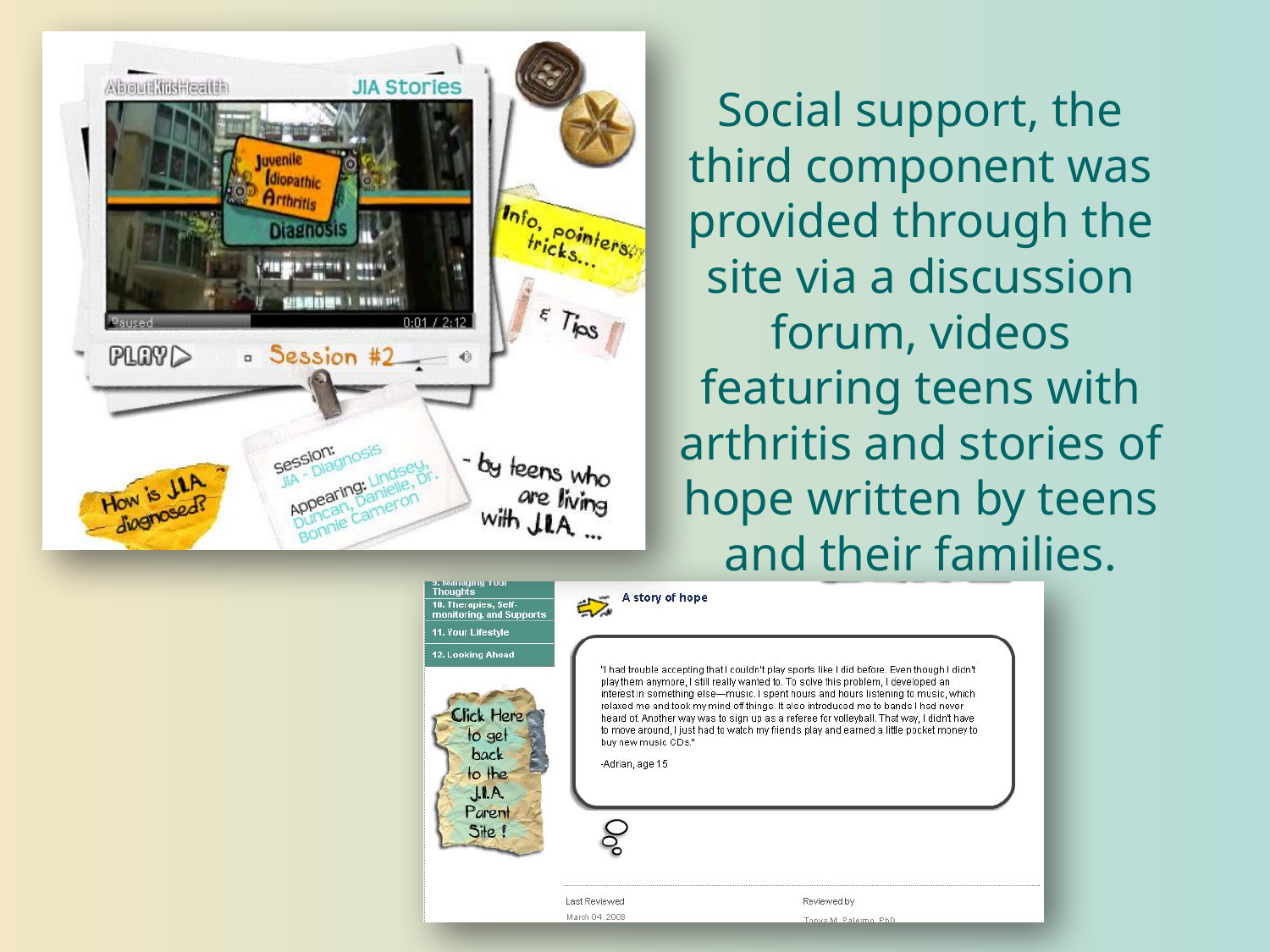

Social support, the third component was provided through the site via a discussion forum, videos featuring teens with arthritis and stories of hope written by teens and their families.

## Slide 5
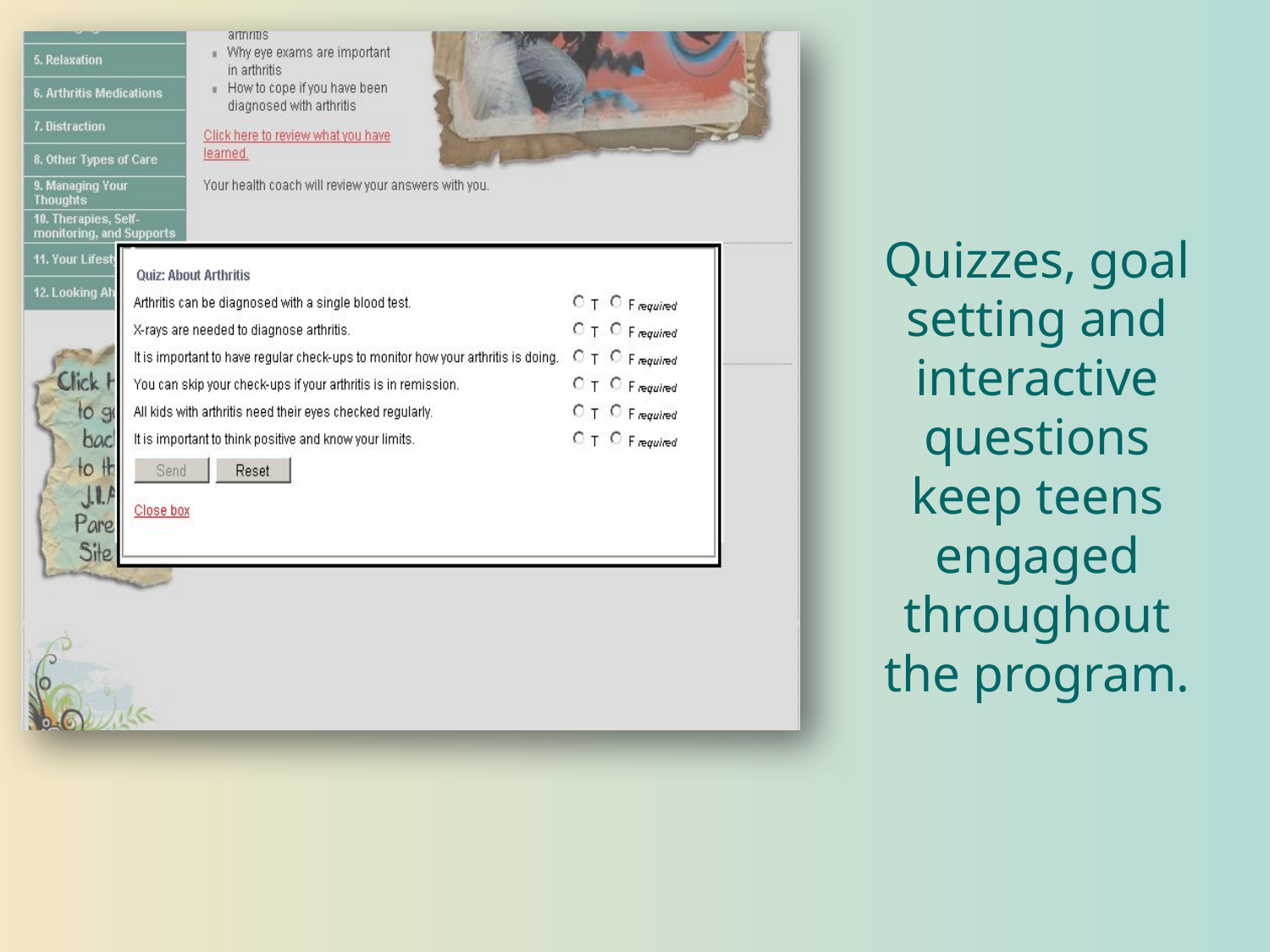

Quizzes, goal setting and interactive questions keep teens engaged throughout the program.
